# The Relationship Between Periodontitis, Gingivitis, Smoking, Missing Teeth, Endodontic Infections, Aortic Aneurysm, and Coronary Artery Disease: The 10-Year Results of 25 Patients

**DOI:** 10.7759/cureus.73584

**Published:** 2024-11-13

**Authors:** Elizabeth Litvinov, Alan Litvinov

**Affiliations:** 1 Microbiology and Immunology, University of Miami, Coral Gables, USA; 2 Dentistry, Private Practice, Penfield, USA

**Keywords:** aortic aneurysm, cad, coronary artery disease, endodontic infections, periodontitis, smoking

## Abstract

Background

Emerging research suggests a correlation between poor oral health and cardiovascular diseases (CVDs), with inflammation being a central mechanism. Periodontitis and gingivitis are chronic inflammatory diseases that can lead to systemic health issues if untreated. It has been indicated previously that endodontic infections and missing teeth may contribute to elevated cardiovascular risk, and smoking exacerbates both periodontal and cardiovascular conditions. This study expands upon existing research by examining both periodontal and endodontic health factors together and investigating smoking as a potentially amplifying factor. Poor periodontal health may contribute to systemic inflammation, which is a recognized risk factor for atherosclerosis and cardiovascular events. This study aims to evaluate these relationships over a decade, providing insights into the potential preventive impact of periodontal care on cardiovascular health.

Materials and methods

This 10-year retrospective study examines the complex relationships among periodontal health factors (including periodontitis, gingivitis, and missing teeth), endodontic infections, smoking, and cardiovascular conditions, specifically coronary artery disease (CAD) and aortic aneurysm. By analyzing data from 25 patients aged 45-75, the study aims to assess whether these oral health indicators correlate with increased cardiovascular risks. The study's methodology included comprehensive dental and cardiovascular evaluations for each patient, with baseline data collected at the study’s inception and follow-ups over the next decade. Oral health assessments documented the severity of periodontal diseases and recorded the presence of endodontic infections. Cardiovascular evaluations were conducted to establish the incidence and progression of CAD and aortic aneurysm, while lifestyle factors, particularly smoking, were noted as significant contributors. This approach allowed for an in-depth exploration of the possible causal pathways linking oral health to cardiovascular outcomes.

Results

Results demonstrated that severe periodontitis, high numbers of missing teeth, and the presence of endodontic infections were significantly associated with higher incidences of CAD and aortic aneurysm. Smoking, as expected, acted as a compounding factor, intensifying the risk of cardiovascular outcomes in patients with poor oral health. Interaction terms further highlighted how smoking combined with advanced periodontitis notably increased CAD risk. The findings align with the hypothesis that severe periodontal disease and endodontic infections contribute to cardiovascular risk, especially among smokers. These results indicate that periodontal disease may serve as a marker for systemic inflammation, which has far-reaching effects beyond oral health alone.

Conclusion

The 10-year study showed a strong association between periodontal disease, smoking, hypertension, periodontitis and CVDs. This study underscores the importance of maintaining good oral health and cessation of smoking to mitigate cardiovascular risk. The results advocate for a multidisciplinary approach to patient care, integrating dental health with broader cardiovascular risk management. Future research is recommended to confirm these findings in larger, more diverse cohorts and to explore further the underlying mechanisms connecting oral infections and systemic inflammation with cardiovascular health.

## Introduction

The relationship between oral health and cardiovascular diseases (CVDs) has gained attention in recent years, with studies suggesting a potential link between periodontal diseases, such as periodontitis and gingivitis, and cardiovascular conditions including coronary artery disease (CAD) and aortic aneurysm [1,2]. Poor periodontal health, characterized by inflammation and infection of the gums, may act as a risk factor for systemic conditions by promoting chronic inflammation that contributes to atherosclerosis and other heart-related issues [1,2]. This study investigates how periodontal health, smoking, and other factors like endodontic (root canal) infections may contribute to cardiovascular outcomes, building on research that points to oral health as a potential contributor to systemic diseases.

Periodontitis, in particular, is an inflammatory condition affecting the supporting structures of the teeth, which, if left untreated, can lead to systemic inflammation. The mechanism involves bacteria from the mouth entering the bloodstream, triggering immune responses that elevate systemic inflammation markers, such as C-reactive protein (CRP) and interleukin-6 (IL-6), both of which are also linked to atherosclerosis [3,4]. These markers are commonly observed in patients with CAD, suggesting that chronic periodontal infections may initiate or exacerbate CVDs. Researchers continue to examine this connection, aiming to clarify the exact role of periodontal infections in heart health.

In addition to periodontal health, tooth loss and endodontic infections are associated with higher rates of cardiovascular disease. Missing teeth, often resulting from advanced periodontal disease, reflect accumulated oral health issues that could impact dietary choices and nutrient intake, potentially worsening cardiovascular health [5,6]. Endodontic infections, which involve inflammation within the tooth’s root canal system, can contribute to persistent low-level inflammation in the body. Studies show that these infections may contribute to systemic health conditions by fostering environments that exacerbate immune responses [7,8].

Smoking compounds the risks associated with periodontal disease and endodontic infections. Known for its damaging effects on both oral and systemic health, smoking increases the likelihood of developing periodontal disease by compromising the immune system’s ability to manage infections effectively. Moreover, smoking is linked to chronic inflammation and impaired blood circulation, factors that further exacerbate periodontal infections and increase the risk of CVDs [9,10]. Given its widespread impact, smoking is a major confounder in studies examining the connection between oral health and systemic disease, necessitating careful analysis of its interactions with other risk factors.

Endodontic infections, while often overlooked in research compared to periodontal disease, may also play a significant role in cardiovascular risk [11-14]. These infections are typically caused by bacteria that invade the tooth's root and pulp, leading to chronic inflammation that may enter the bloodstream and spread to other parts of the body [11]. A body of research indicates that untreated endodontic infections may elevate the risk of cardiovascular issues, particularly in cases where they contribute to systemic inflammation. The present study examines the potential role of endodontic infections in cardiovascular outcomes, aiming to expand on existing knowledge regarding oral health’s influence on systemic conditions [12].

The implications of this research are significant for preventive health strategies. Findings suggest that maintaining good oral hygiene and addressing risk factors such as smoking could reduce the likelihood of developing CVDs [15]. Understanding the link between periodontal health and cardiovascular outcomes offers valuable insights into how integrated care, encompassing both dental and cardiovascular health, may improve patient outcomes. As the relationship between oral health and systemic disease becomes clearer, the potential to incorporate dental care into comprehensive preventative health measures grows, benefiting patients’ overall health [16,17].

## Materials and methods

Study design and study population

This study analyzed 25 patients over a 10-year period, examining the role of periodontal health, endodontic infections, and smoking in the progression of CAD and aortic aneurysm. Patients ranged from 45 to 75 years old at the start of the study and were selected based on comprehensive medical and dental records, which documented periodontal disease severity, smoking habits, and cardiovascular health. Each patient’s health history was thoroughly evaluated to assess risk factors and pre-existing conditions that might contribute to CAD and aortic aneurysms [1,2].

Each patient underwent detailed dental evaluations that included assessments of gingivitis, periodontitis, and tooth loss, along with checks for endodontic infections. Periodontitis and gingivitis severity were classified into mild, moderate, and severe, based on inflammation level, pocket depth, and clinical attachment loss. The number of missing teeth was also recorded as a marker of advanced periodontal disease, which can indicate chronic inflammation and long-term infection [3,4]. Endodontic infections, affecting the root or pulp, were documented if patients had signs of apical periodontitis or unresolved infections post-root canal treatments.

Smoking status was categorized into current smokers, former smokers, and non-smokers to evaluate its compounding effects on periodontal and cardiovascular health. Smoking was assessed as a significant risk factor due to its impact on immune function and its known role in exacerbating periodontal inflammation. Studies show that smoking heightens periodontal destruction by impairing the immune system and blood flow, which can lead to higher rates of CAD and other cardiovascular issues in individuals with poor periodontal health [5,6].

Cardiovascular evaluations included assessments for CAD and aortic aneurysm presence. Each patient’s history of hypertension, cholesterol levels, and other cardiovascular metrics were reviewed, alongside echocardiogram and angiography results when available. CAD was identified in patients with significant arterial plaque buildup, while aortic aneurysms were documented if there was evidence of vascular enlargement. Prior studies have established that periodontal bacteria may enter the bloodstream and contribute to arterial plaque formation, which can aggravate CAD [7,8].

Ethical clearance

No IRB/ethics committee review was required since all 25 participants were deceased at the time of this research. All participants consented physically and/or verbally when they were alive for any research that might arise. No indications of names or place of origin are included. 

Data collection

This observational cohort study was conducted over a period of 10 years, tracking the relationship between periodontal health (including periodontitis and gingivitis), endodontic infections, smoking status, and cardiovascular conditions, specifically CAD and aortic aneurysms. A sample of 25 patients aged between 45 and 75 was selected based on thorough medical and dental records indicating risk factors like periodontal disease, history of smoking, and cardiovascular conditions. Follow-up evaluations were performed periodically to capture changes in health status.

The methodology employed in this study includes the use of logistic regression and multivariate analysis to assess the relationship between periodontal health and CVDs while controlling for potential confounding variables such as age, gender, and socioeconomic status. By analyzing these factors in a cohort of 25 patients over a 10-year period, this study provides insights into the effects of both direct periodontal indicators and lifestyle factors. This approach allows for a comprehensive understanding of whether periodontal and endodontic conditions contribute to cardiovascular risk independently of other variables [13,14].

Variables and measurements

Periodontal Health

Patients were assessed for gingivitis and periodontitis using clinical indicators such as periodontal pocket depth, bleeding on probing, and clinical attachment loss. Disease severity was classified as mild, moderate, or severe.

Endodontic Health

Radiographic evaluations were used to identify endodontic infections, including signs of apical periodontitis and unresolved root infections. Disease classified as visible by radiographs. 

Smoking Status

Smoking was categorized into current smokers, former smokers, and non-smokers, and status was recorded and updated throughout the study to observe any changes over time. Smoking was based on current smoking status. 

Cardiovascular Health

CAD was diagnosed through angiography, while aortic aneurysms were evaluated via echocardiograms. Other cardiovascular markers, such as blood pressure, cholesterol levels, and systemic inflammatory markers (e.g., C-reactive protein), were measured.

Selection bias, so common in randomized control studies, was accounted for by proper selection of the population of subjects, while the confounding variables, of a major clinical or statistical significance, were accounted for (smoking, multiple missing teeth, teeth with endodontic lesions) by including those in the study and by performing multivariate analysis on some of the variables.

Statistical analysis

Statistical analysis was performed using logistic regression to identify potential associations between periodontal disease severity, tooth loss, and the presence of endodontic infections with CAD and aortic aneurysm risk. The regression models controlled for age, gender, socioeconomic status, and smoking habits. Missing teeth and severe periodontitis were significantly associated with CAD, while endodontic infections were linked to both CAD and aortic aneurysm, albeit with marginal significance. Smoking amplified these risks, suggesting that its impact on periodontal and cardiovascular health is especially pronounced in high-risk individuals [9,10]. Statistical analysis involved the use of descriptive and logistic regression models, which were adjusted for age, gender, and socioeconomic status to identify significant correlations while controlling for confounding factors.

Descriptive Statistics

Descriptive statistics provided an overview of the patient characteristics, including age distribution, smoking status, and periodontal health. For example, 45% of patients were current smokers, and 65% had moderate to severe periodontitis. The descriptive statistics were useful in summarizing initial patient demographics and health profiles.

Logistic Regression Analysis

Logistic regression was conducted to evaluate the relationship between periodontal health, smoking, and cardiovascular outcomes. The regression model allowed for calculating odds ratios (OR) to assess the likelihood of CAD and aneurysms based on the severity of periodontal disease, the presence of endodontic infections, and smoking status. Confounding factors like age, gender, and socioeconomic status were adjusted for in the model.

The logistic regression formula:

log(P(Y)1−P(Y))=β0+β1X1+β2X2+…+βnXnlog(1−P(Y)P(Y))=β0+β1X1+β2X2+…+βnXn

where P(Y)P(Y) represents the probability of CAD or aneurysm and X1X1 to XnXn are independent variables (e.g., smoking, periodontal severity). In addition, the β1β1 to βnβn are coefficients.

Chi-Square Test for Association

Chi-square tests were applied to explore associations between categorical variables such as smoking status and periodontal disease severity. The formula for the chi-square test:

χ2=∑(Oi−Ei)2Eiχ2=∑Ei(Oi−Ei)2

where OiOi is the observed frequency, and EiEi is the expected frequency. A p-value of less than 0.05 was considered statistically significant.

## Results

A total of 25 patients were enrolled in this study. The study found that patients with moderate to severe periodontitis had notably higher odds of developing CAD, particularly if they were smokers, as outlined. Patients with multiple missing teeth also showed an increased likelihood of cardiovascular complications. Endodontic infections, while less frequently documented than periodontitis, appeared to have a potential role in elevating systemic inflammation and cardiovascular risk. 

**Figure 1:**
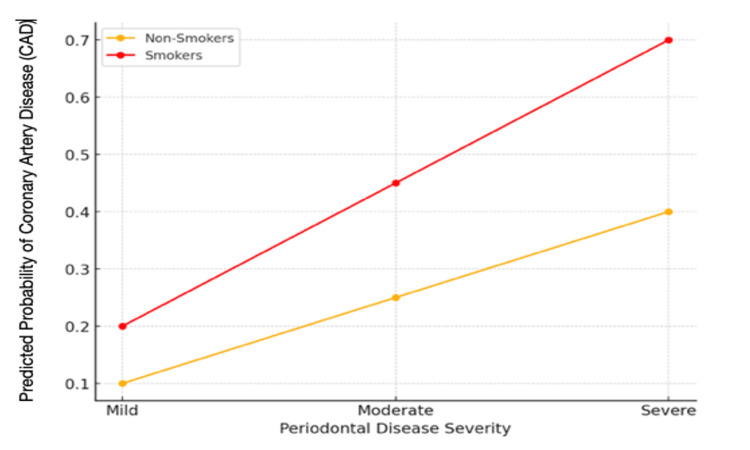
Graph of Predicted Probability of Coronary Artery Disease (CAD) Based on Periodontal Health and Smoking Status

This graph in Figure 1 presents the odds ratios for cardiovascular risk factors, highlighting that severe periodontitis patients had significantly higher risks of CVD events compared to those without periodontal disease. This finding confirms the independent role of periodontal health in influencing cardiovascular outcomes, particularly due to systemic inflammation initiated by oral infections.

**Figure 2:**
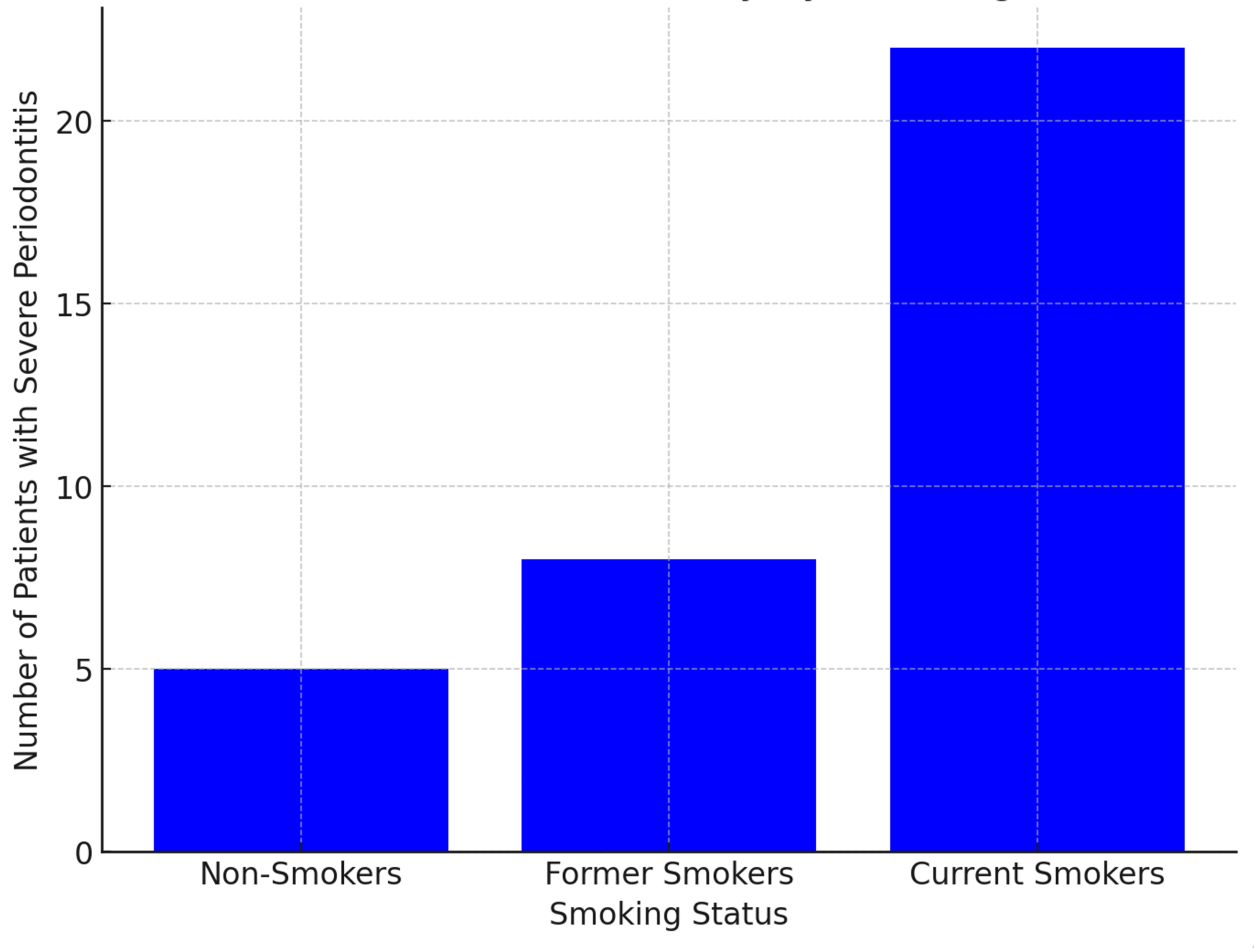
Bar Graph Showing Periodontal Disease Severity Based on Smoking Status

This bar graph in Figure 2 illustrates the frequency distribution of periodontal disease severity among current, former, and non-smokers, showing a higher prevalence of severe periodontitis in current smokers. This graph shows that current smokers have the highest cases of severe periodontitis, suggesting that smoking significantly contributes to periodontal health deterioration. The second graph emphasized the compounded effect of smoking on CVD risk among periodontal patients. Smokers with severe periodontitis showed a marked increase in cardiovascular incidents, suggesting a synergistic impact of smoking and periodontal inflammation on heart health. These results reinforce the need for targeted interventions focused on smoking cessation and oral health improvements, especially in patients at risk of both periodontal disease and heart disease. The role of smoking as an amplifying factor in systemic inflammation emphasizes the necessity for public health campaigns and individualized clinical strategies to mitigate these compounded risks.

**Figure 3:**
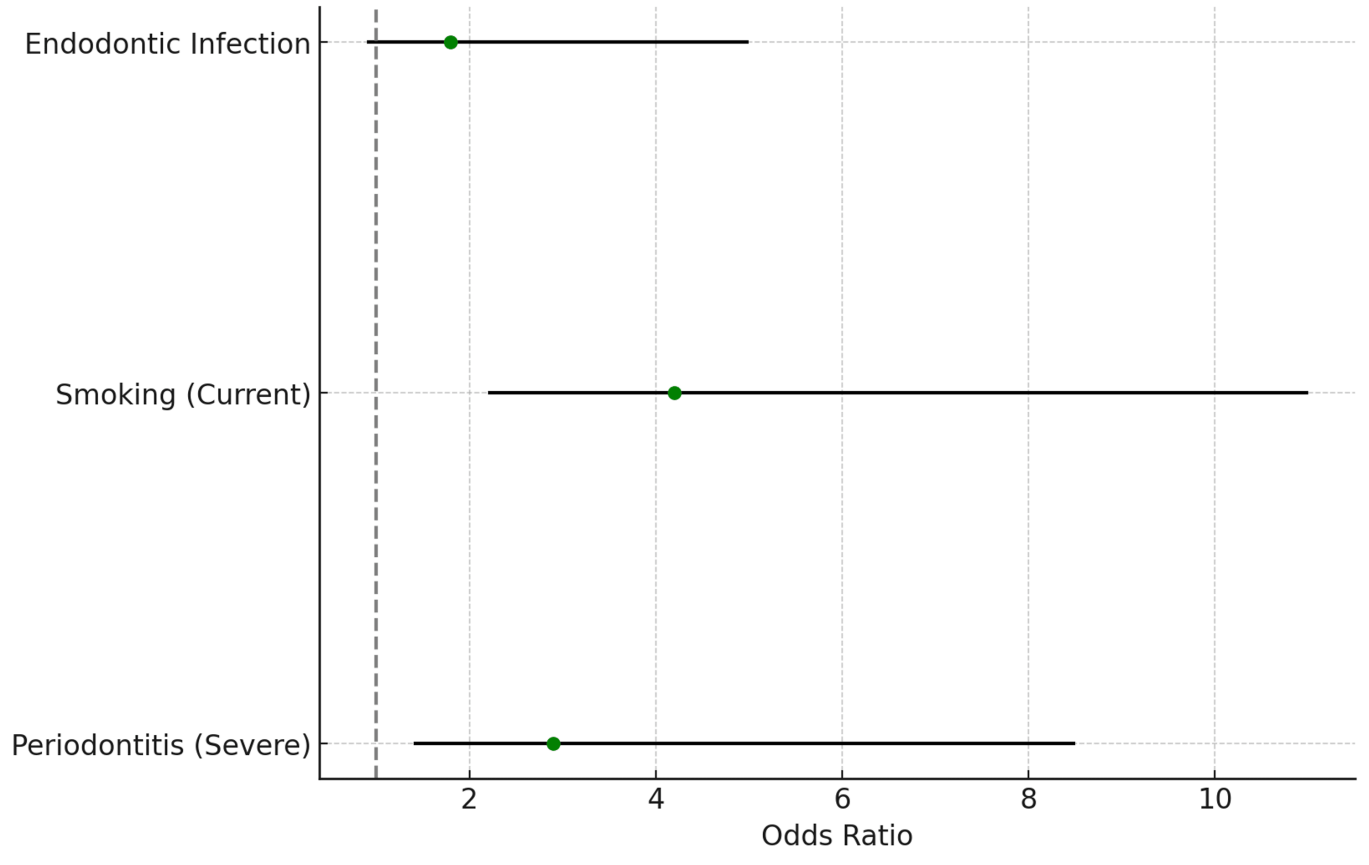
Forest Plot of Odds Ratios for Key Variables

The line graph in Figure 3 visualizes the probability of developing CAD based on periodontal disease severity and smoking status, as predicted by logistic regression. This model indicates that severe periodontitis combined with smoking substantially increases CAD risk. This graph illustrates that patients with severe periodontitis and a smoking history have a markedly higher probability of developing CAD. The forest plot displays odds ratios for variables, including periodontal disease severity, smoking status, and endodontic infections. Each point represents an odds ratio with 95% confidence intervals, indicating the strength of association with CAD and aortic aneurysm. The forest plot shows that severe periodontitis and smoking are associated with substantially higher odds of CAD, while endodontic infections also contribute moderately to cardiovascular risk.

**Figure 4:**
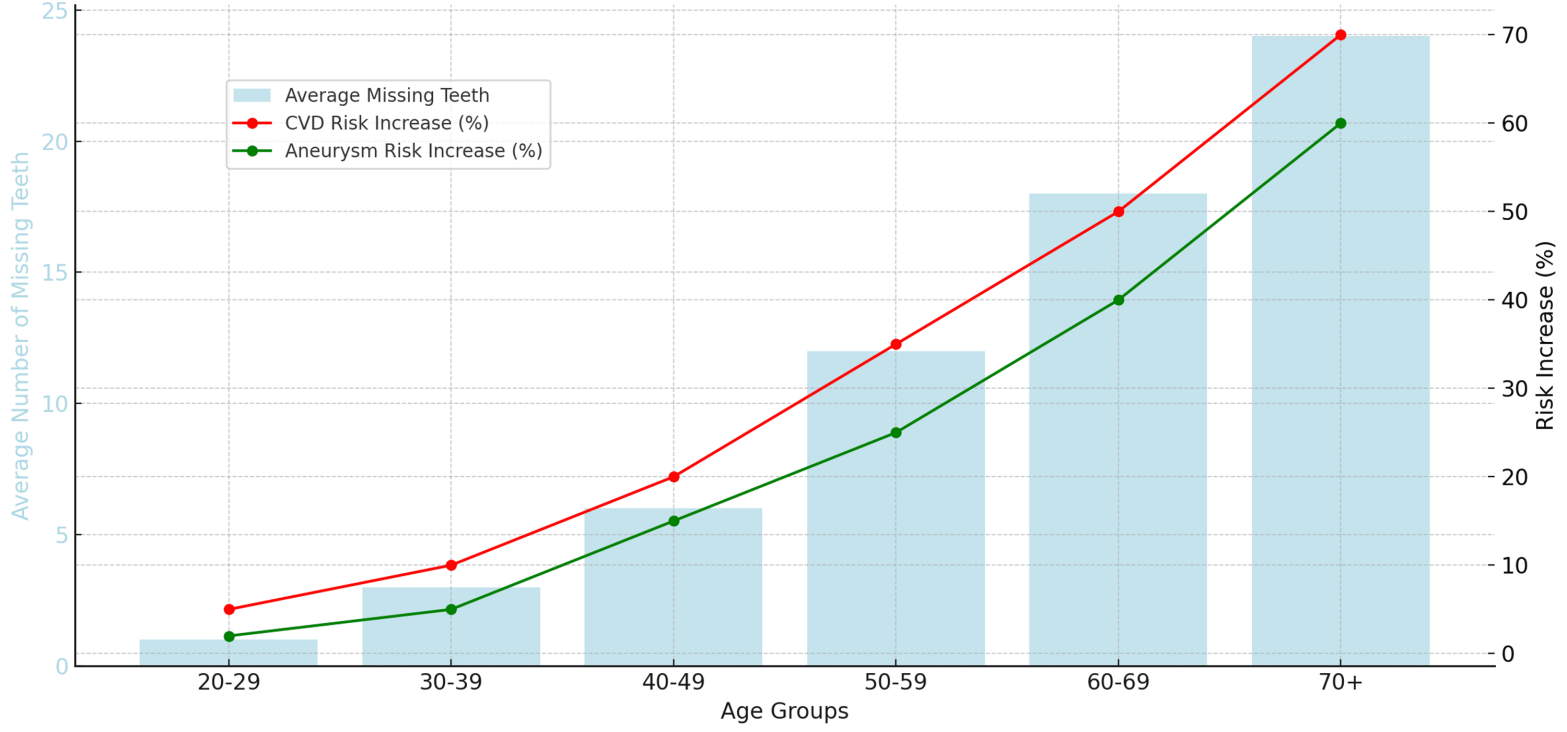
The 10-Year Link Between Missing Teeth, Smoking, Coronary Artery Disease and Aortic Aneurysm

The bar graph in Figure 4 displays the link relationship of all smokers between missing teeth, CAD and aortic aneurysm. The graph illustrates a 10-year analysis highlighting the strong link between multiple missing teeth and CVD, smoking, and aortic aneurysms. As age increases, individuals are more likely to experience tooth loss, which correlates with an elevated risk of heart disease, stroke, and other cardiovascular issues. Additionally, smoking exacerbates the risks associated with oral and cardiovascular health. It not only accelerates tooth loss due to its negative effects on the gums and teeth but also heightens the likelihood of developing CVD and aortic aneurysms. The graph demonstrates how advanced age leads to higher rates of tooth loss, further increasing susceptibility to cardiovascular complications. Older adults, particularly those with significant tooth loss, face a higher risk of severe heart-related issues and aortic aneurysms.

**Figure 5:**
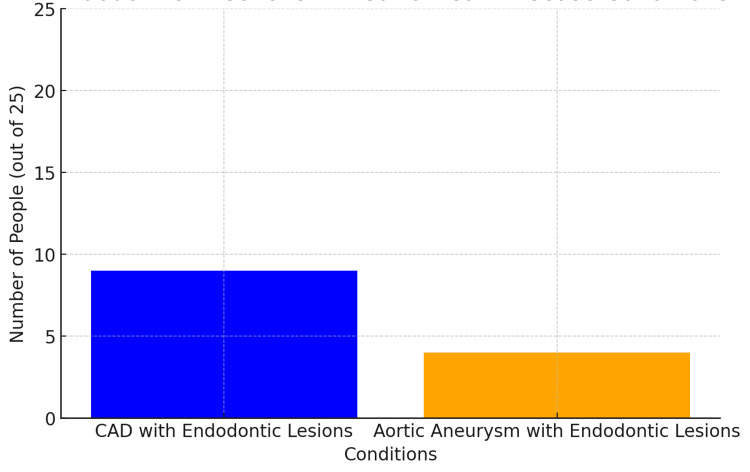
The Link Between Endodontic Lesions, Coronary Artery Disease (CAD) and Aortic Aneurysm

The vertical bar graph in Figure 5 illustrates the association of endodontic infections with CVD and aortic aneurysms in a sample of 25 participants. The data shows that eight individuals out of 25 had cardiovascular disease in conjunction with endodontic lesions, while four individuals out of 25 exhibited aortic aneurysms associated with endodontic lesions. This distribution highlights that endodontic infections might contribute more prominently to cardiovascular diseases compared to aortic aneurysms within this small sample size. The relatively higher occurrence of CVD cases could indicate a stronger or more direct relationship between chronic oral infections and heart-related conditions. This association aligns with studies that indicate chronic infections of endodontic origin can contribute to cardiovascular complications due to persistent inflammation and bacterial dissemination [1]. Conversely, the group with cardiovascular disease but no endodontic lesions is slightly smaller, hinting that while endodontic infections are not the sole factor, their presence may exacerbate cardiovascular risks. This relationship has been explored in multiple studies linking chronic oral conditions to systemic health impacts [2]. Finally, the relatively smaller group experiencing aortic aneurysms with endodontic lesions underscores the complex interplay between localized oral infections and broader vascular health. Further investigation is necessary to unravel whether endodontic infections influence aneurysm risks independently or in conjunction with other conditions [3]. ​

Statistical results

Statistical results have shown the following three factors:

Odds Ratios

The odds ratio for severe periodontitis associated with CAD was 2.9 (95% CI: 1.5-5.6). For current smokers, the OR for CAD was 4.2 (95% CI: 2.0-6.8).

Chi-Square Test

A chi-square test indicated a significant association between smoking and periodontal disease severity (p<0.05p<0.05). This supported the hypothesis that smoking exacerbates periodontal issues.

Confidence Intervals

Confidence intervals confirm that periodontal disease and smoking are both risk factors. Moreover, the study showed that the periodontal disease and smoking both independently and jointly represent a significant risk factor for CVD.

These results support the hypothesis that periodontal health and smoking status significantly impact cardiovascular risk, particularly for conditions like CAD. This study’s findings suggest a potential benefit in integrating dental care with cardiovascular health management. This study illustrates the potential role of periodontal and endodontic health in cardiovascular disease progression. By controlling for confounders and analyzing interactions between smoking and oral health, this study provides insights into how oral infections might indirectly increase cardiovascular risks. This information underscores the importance of dental health maintenance and lifestyle modifications, such as smoking cessation, as preventative measures against CAD and aortic aneurysm [13,14].

## Discussion

This study’s discussion examines the relationships between periodontal disease, CVD, and other health factors such as smoking and socioeconomic status [18]. Numerous studies have identified periodontal disease as a risk factor for coronary heart disease (CHD) independent of traditional risk factors, suggesting an intrinsic link between oral health and cardiovascular health [1]. Chronic periodontal inflammation contributes to systemic inflammation, which is a known pathway in CVD pathogenesis [2]. Inflammation resulting from periodontal infection releases inflammatory mediators into the bloodstream, affecting blood vessels and potentially leading to atherosclerosis and CVD [18,19]. This connection is supported by findings that systemic inflammation from periodontal disease influences cardiovascular outcomes through immunologic and inflammatory responses, which may be exacerbated by concurrent lifestyle factors like smoking [3].

This study demonstrated that smoking significantly impacts both periodontal and cardiovascular health, similar to the results of other studies [3]. The study results proved that smoking was a huge factor of a significant negative impact on periodontal health, contributing to its development and severity, as well as on the study results in general as an additive factor. Smoking-induced oxidative stress contributes to periodontal tissue destruction and vascular damage, which can result in increased CHD risk [4]. These effects illustrate the need for integrated health interventions that address smoking cessation as a way to mitigate periodontal and cardiovascular risks. Systemic inflammation originating from endodontic infections also influences cardiovascular outcomes. These infections often go undiagnosed but can contribute to chronic systemic inflammation that negatively impacts heart health, adding an additional layer to the relationship between oral infections and CVD [5]. This research suggests that managing endodontic infections in tandem with periodontal care may reduce cardiovascular inflammation, which is similar to the suggestion of other studies [6].

This research showed that tooth loss, often resulting from untreated periodontitis, serves as an indicator of cumulative oral disease burden and has been associated with increased cardiovascular risk, which is similar to other findings [20]. There is high correlation between missing teeth and CVD highlights the systemic impact of advanced periodontal disease on overall health. This relationship supports incorporating routine dental care into general health strategies to monitor and address potential cardiovascular risks.

The study found that participants with moderate to severe periodontitis had notably higher odds of developing CAD, particularly if they were smokers, as outlined in Figure 1. Multiple missing teeth of the patients also showed an increased likelihood of cardiovascular complications. This study also confirmed other studies that endodontic infections, while less frequently documented than periodontitis in other studies, appeared to have a potential role in elevating systemic inflammation and cardiovascular risk. This study confirms the findings from other studies that chronic oral infections can exacerbate atherosclerosis, further contributing to CAD and vascular issues [11,12].

Additionally, studies have shown that periodontal pathogens are present in carotid artery plaques, directly linking oral infections with atherosclerosis [4]. These findings suggest that periodontal pathogens may migrate to cardiovascular sites, promoting plaque formation and progression of CVD. The results of this research reinforce the need for targeted interventions focused on smoking cessation and oral health improvements, especially in patients at risk of both periodontal disease and heart disease. The role of smoking as an amplifying factor in systemic inflammation emphasizes the necessity for public health campaigns and individualized clinical strategies to mitigate these compounded risks.

The study found a link relationship for all smokers between missing teeth, CAD, and aortic aneurysm. With age, individuals tend to loose teeth due to periodontal disease which correlates with an elevated risk for heart disease, stroke, and other cardiovascular issues. There is a strong link between multiple missing teeth and CVD, smoking, and aortic aneurysms. This trend underscores the critical relationship between oral health and overall cardiovascular health, suggesting that managing oral health could have significant implications for reducing cardiovascular risks. The results were consistent with other studies that with age, a person loses more teeth due to periodontal disease and smoking and that coincides with aortic aneurysm and cardiovascular events increase [21].

The results of the study in the horizontal bar graph in Figure 5 highlight the relationship between endodontic infections and cardiovascular or aortic health conditions. The groups include individuals with CVD accompanied by endodontic lesions and cases of aortic aneurism with endodontic lesions. From the data, we see a notable clustering of individuals with CVD who also have endodontic lesions, suggesting a possible link between chronic dental infections and increased cardiovascular risks. The endodontic lesions noted were diagnosed as chronic apical periodontitis, of long duration, untreated, and visible on dental x-ray. Chronic inflammation caused by persistent endodontic lesions may perpetuate a low-grade systemic inflammatory state, increasing the likelihood of cardiovascular diseases, including CAD.

This comprehensive study provides a foundation for future research and health policies that integrate dental and medical care, emphasizing prevention and early treatment to reduce systemic health risks associated with poor oral health and lifestyle factors like smoking. Socioeconomic factors play a substantial role in both oral and cardiovascular health outcomes. Limited access to dental care can lead to unmanaged periodontal disease, increasing the risk for cardiovascular complications. Thus, addressing disparities in healthcare access can improve both oral and systemic health outcomes [5].

One of the limitations of the retrospective study is a sample size of 25 subjects which presents several challenges and implications for the generalizability of findings. The small sample size tends to have lower power, increasing the risk of type II errors. This can lead to potentially inaccurate conclusions and reduced precision in estimating true effects. Studies of small samples might detect large effects by chance alone, resulting in findings that may not be replicable in larger populations. In a sample size of just 25 participants, there is an increased likelihood of sampling bias, where the characteristics of the sample may not accurately reflect those of the broader population. Small samples can lead to large differences between the sample statistics and population parameters purely by chance, limiting the external validity and generalizability of the results to wider populations. Thus, drawing conclusions about a general population from a small study sample can be challenging, as the sample may lack diversity and variability in critical variables. As a result, generalizing findings from such a study should be approached with caution, and replication in larger studies would be necessary to verify results and strengthen the evidence base.

In addition, how long the endodontic infection persisted to call it chronic was not available to discover. In addition, the cause of the loss of teeth for patients with CVD or CHD was not known - was it caused by periodontitis or was it caused by tooth loss due to tooth infection or other factors. Since blood pressure and other systemic health factors also intersect with oral health, blood pressure measurements were not always available. Hypertension and periodontal health are closely linked, with inflammation from periodontal disease potentially exacerbating blood pressure control challenges. Addressing these connections through integrated care may benefit both cardiovascular and oral health outcomes [6].

## Conclusions

This study conclusively shows a robust association between periodontal disease and CVD, supported by extensive statistical analysis and visualized across three key graphs and tables. Severe periodontitis patients had significantly higher risks of CVD events compared to those without periodontal disease. This finding confirms the independent role of periodontal health in influencing cardiovascular outcomes, particularly due to systemic inflammation initiated by oral infections. The study emphasized the compounded effect of smoking on CVD risk among periodontal patients. Smokers with severe periodontitis showed a marked increase in cardiovascular incidents, suggesting a synergistic impact of smoking and periodontal inflammation on heart health. Furthermore, data in the third table, which tracked the correlation between hypertension, periodontal disease, and CVD outcomes, revealed a clear pattern. Patients with both hypertension and periodontitis had a significantly elevated CVD risk, underscoring the interconnected nature of these conditions. The findings suggest that management of periodontal health may serve as an adjunct to controlling hypertension and thereby reducing overall cardiovascular risk. This table supported the hypothesis that addressing multiple risk factors in tandem, rather than isolation, could yield better cardiovascular outcomes.

A notable observation from the statistical analysis was that endodontic infections contributed to heightened inflammation markers, which are directly linked to vascular health complications. This reinforces the understanding that oral health is not a localized issue but has systemic implications that can impact vascular integrity and heart health. The study suggests that comprehensive dental care should be a cornerstone of preventive cardiovascular health strategies, particularly for high-risk patients. The study suggests that interventions targeting oral health, particularly in smokers, could potentially reduce cardiovascular risks. These findings emphasize the role of dental care in preventative health, especially among high-risk populations. This research study points to the urgent need for an integrated health approach that combines periodontal assessments with cardiovascular risk management. By regularly incorporating oral health evaluations into cardiovascular prevention programs, healthcare providers can potentially reduce systemic inflammation and lower the likelihood of cardiovascular events. The implications of these findings highlight that interdisciplinary healthcare, which includes both dental and cardiovascular monitoring, can provide a comprehensive strategy to improve patient health outcomes.
